# TRansfusion strategies in Acute brain INjured patients (TRAIN): a prospective multicenter randomized interventional trial protocol

**DOI:** 10.1186/s13063-022-07061-7

**Published:** 2023-01-07

**Authors:** Fabio Silvio Taccone, Rafael Badenes, Carla Bittencourt Rynkowski, Pierre Bouzat, Anselmo Caricato, Pedro Kurtz, Kirsten Moller, Manuel Quintana Diaz, Mathieu Van Der Jagt, Walter Videtta, Jean-Louis Vincent

**Affiliations:** 1 Department of Intensive Care, Route de Lennik, Erasme Hospital, Université Libre de Bruxelles, 808, 1070 Brussels, Belgium; 2 Hospital Nacional Professor Alejandro Posadas, Buenos Aires, Argentina; 3 Department of Anesthesiology and Surgical-Trauma ICU, Hospital Clínic Universitari de Valencia, University of Valencia, Valencia, Spain; 4 Intensive Care Unit of Cristo Redentor Hospital, Porto Alegre, Brazil; 5 Intensive Care Unit, Hospital Ernesto Dornelles, Porto Alegre, Brazil; 6 Université Grenoble AlpesInserm, U1216, CHU Grenoble Alpes, Grenoble Institut Neurosciences, Grenoble, France; 7 Institute of Anesthesiology and Intensive Care, Catholic University School of Medicine, Rome, Italy; 8 Department of Intensive Care Medicine, DOr Institute of Research and Education, Rio de Janeiro, Brazil; 9 Department of Neurointensive Care, Instituto Estadual Do Cerebro Paulo Niemeyer, Rio de Janeiro, Brazil; 10 Department of Neuroanaesthesiology, Neuroscience Centre, Copenhagen University, Hospital Rigshospitalet, Copenhagen, Denmark; 11 Department of Clinical Medicine, Faculty of Health and Medical Sciences, University of Copenhagen, Copenhagen, Denmark; 12 Department of Intensive Care Medicine, Hospital Universitario de La Paz, Madrid, Spain; 13 Department of Intensive Care Adults, Erasmus MC – University Medical Center Rotterdam, Rotterdam, The Netherlands

**Keywords:** Anemia, Brain perfusions, Tissue hypoxia, Brain injury, Outcome, Clinical study, Randomized controlled trial

## Abstract

**Background:**

Although blood transfusions can be lifesaving in severe hemorrhage, they can also have potential complications. As anemia has also been associated with poor outcomes in critically ill patients, determining an optimal transfusion trigger is a real challenge for clinicians. This is even more important in patients with acute brain injury who were not specifically evaluated in previous large randomized clinical trials. Neurological patients may be particularly sensitive to anemic brain hypoxia because of the exhausted cerebrovascular reserve, which adjusts cerebral blood flow to tissue oxygen demand.

**Methods:**

We described herein the methodology of a prospective, multicenter, randomized, pragmatic trial comparing two different strategies for red blood cell transfusion in patients with acute brain injury: a “liberal” strategy in which the aim is to maintain hemoglobin (Hb) concentrations greater than 9 g/dL and a “restrictive” approach in which the aim is to maintain Hb concentrations greater than 7 g/dL. The target population is patients suffering from traumatic brain injury (TBI), subarachnoid hemorrhage (SAH), or intracerebral hemorrhage (ICH). The primary outcome is the unfavorable neurological outcome, evaluated using the extended Glasgow Outcome Scale (eGOS) of 1–5 at 180 days after the initial injury. Secondary outcomes include, among others, 28-day survival, intensive care unit (ICU) and hospital lengths of stay, the occurrence of extra-cerebral organ dysfunction/failure, and the development of any infection or thromboembolic events. The estimated sample size is 794 patients to demonstrate a reduction in the primary outcome from 50 to 39% between groups (397 patients in each arm). The study was initiated in 2016 in several ICUs and will be completed in December 2022.

**Discussion:**

This trial will assess the impact of a liberal versus conservative strategy of blood transfusion in a large cohort of critically ill patients with a primary acute brain injury. The results of this trial will help to improve blood product and transfusion use in this specific patient population and will provide additional data in some subgroups of patients at high risk of brain ischemia, such as those with intracranial hypertension or cerebral vasospasm.

**Trial registration:**

ClinicalTrials.gov NCT02968654.

**Supplementary Information:**

The online version contains supplementary material available at 10.1186/s13063-022-07061-7.

## Administrative information

The numbers in brackets at each paragraph in this protocol refer to SPIRIT checklist item numbers. The order of the items has been modified to group similar items (see http://www.equator-network.org/reporting-guidelines/spirit-2013-statement-defining-standard-protocol-items-for-clinical-trials/). The SPIRIT Checklist has been submitted as Supplemental Material.

**Table:** 

Title	TRansfusion strategies in Acute brain INjured patients (TRAIN): A Prospective Multicenter Randomized Interventional Study
Trial registration	Hôpital Erasme register number P2015/327 Clinicaltrials.gov register number NCT02968654
Protocol version	3.0
Funding	European Society of Intensive Care (ESICM) La Fondation des Geules Cassées
Author details	Fabio Silvio TACCONE1, Rafael BADENES^2^, Carla BITTENCOURT RYNKOWSKI^3^, Pierre BOUZAT^4^, Anselmo CARICATO^5^, Pedro KURTZ^6^, Kirsten MOLLER^7^, Manuel QUINTANA DIAZ^8^, Mathieu VAN DER JAGT^9^, Walter VIDETTA^10^, Jean-Louis VINCENT^1^ ^1^Department of Intensive Care, University Hospital Brussels, Bruxelles, Belgium
Name and contact information for the trial sponsor	University Hospital Brussels (HUB) Route de Lennik, 808 1070—Bruxelles, Belgium
Role of sponsor	Sponsor has provided support for the ethical submission and data agreement transfers with all participating centres. No additional role for study design, data collection, analysis and writing process is associated with the Sponsor

## Background and rationale

Although red blood cell transfusion (RBCT) can be lifesaving in extreme circumstances, in the absence of life-threatening hemorrhage, the indications for RBCT are somewhat controversial. Blood transfusions have well-recognized problems, including the need to type and cross-match, the potential transmission of diseases, and the development of transfusion-related complications (such as transfusion-related acute lung injury—TRALI—or transfusion-associated circulatory overload—TACO) and immunosuppression [1–3]. However, anemia has also been associated with increased morbidity and mortality among critically ill patients [4, 5]. As such, determining who and when to transfuse in this patient population is thus a challenge and recent years have seen continuing debate and discussion regarding the optimal transfusion “trigger” [6].

In a landmark multicenter Canadian trial, Hebert and colleagues [7] randomized 838 critically ill patients to either a liberal protocol in which transfusions were administered to maintain hemoglobin levels greater than 9 g/dL or a restrictive strategy in which hemoglobin levels were kept between 7 and 9 g/dL. Overall, the 30-day mortality rate was 19% in the restrictive group and 23% in the liberal transfusion group (i.e., non-significant difference), with a significant lower mortality among younger patients and less sick patients, when randomized to the restrictive strategy group. The ABC study [8], an epidemiological survey of 3534 patients conducted in 146 ICUs of West Europe, confirmed an increased mortality rate in transfused patients, even after adjustment for several confounders. In contrast, the analysis from the Sepsis Occurrence in Acutely Ill Patients (SOAP) database (*n* = 3147) found no significant association of RBCT with an increased risk of death after a multivariable analysis and propensity matching [9]. These results had a definite influence on ICU practice, encouraging intensivists to limit the use of transfusions. Moreover, more recent randomized trials have also suggested that a restrictive transfusion policy might be safe and as effective as a more liberal one in critically ill patients [6, 10].

Importantly, most of these studies did not consider the presence of acute brain injury as a specific target population in whom transfusion threshold could be critical. Several observational studies have shown that Hb levels less than 9 g/dL were associated with a poorer outcome in patients with traumatic brain injury (TBI) or subarachnoid hemorrhage (SAH) [11, 12]. On the other hand, the administration of red blood cells (RBC) was also associated with an increased risk of complications or mortality in this setting [13, 14]. A recent meta-analysis showed that studies aimed at comparing two different transfusion thresholds in these patient populations were largely underpowered to identify the best Hb levels [15]. Accordingly, the effects of transfusion need to be better assessed in acute brain injury. One recent randomized clinical trial compared, in a factorial design, the effects of erythropoietin and two Hb transfusion thresholds (7 g/dL vs. 10 g/dL) on neurological recovery after TBI (*n* = 200) [16]. There were no significant differences in the occurrence of favorable neurological outcomes between groups (43% for 7 g/dL and 33% for 10 g/dL, *p* = 0.28). Moreover, there was a higher incidence of thromboembolic events for the transfusion threshold of 10 g/dL (22% vs. 8%; *p* = 0.009). Nevertheless, the number of patients included in the study was relatively small and the two groups of patients showed mean Hb levels much higher than those associated with the treatment arm to which they were randomized.

### Interventions: description

Patients are randomized to two different thresholds of Hb to determine when RBC transfusion should be initiated (< 7 g/dL vs. < 9 g/dL). After randomization, all patients should preferably receive one unit of RBC at a time. Patients randomized to the lower Hb threshold for transfusion receive packed RBC transfusion whenever their Hb concentration is < 7 g/dL; similarly, patients randomized to the higher threshold are transfused whenever their Hb concentration is < 9 g/dL. Transfusion thresholds are maintained until a maximum of 28 days after randomization or hospital discharge/death, whichever occurs first. Daily Hb concentrations are recorded according to local practices; values from blood gas analyses are also allowed during the ICU stay to avoid protocol violation. Patients can be included in the study only once.

#### Interventions: modifications

Protocol violation is defined as one of the following: (a) inability to maintain the daily max Hb values < 9 g/dL in the restrictive group and avoid transfusion until Hb drops below 7 g/dL OR > 9 g/dL in the liberal group for two consecutive days, (b) one or more transfusions given inappropriately (i.e., in contradiction of the assigned trigger level), and (c) error in type/cross-match between donor and recipient.

Crossover to another treatment arm than allocated is allowed based on the decision of the treating physician, such as (1) acute coronary syndrome, requiring a higher Hb level; (2) acute bleeding requiring multiple RBCT; and (3) lack of available blood. The reason for crossover or protocol violation will be mentioned in the electronic case report form (eCRF).

#### Interventions: adherence

Online material was sent before the start of recruitment to the local investigators, research nurses, and treating physicians to explain the study hypothesis and the need for this study. Monthly newsletters have also been sent to promote the study and underline the importance for the adherence to the study protocol and active recruitment.

#### Interventions: concomitant care

No other limitations in concomitant care and interventions have been provided.

#### Explanation for the choice of comparators

In the absence of optimal Hb thresholds to initiate RBCT in this setting, an international survey was conducted to investigate at which Hb level clinicians would initiate RBCT in patients with acute brain injury [17]; among 868 responses, 54% reported an Hb threshold of 7–8 g/dL to initiate RBCT in this setting, although half of these respondents would use a different threshold (i.e., closer to 9 g/dL) in case of systemic and cerebral triggers. Moreover, one small randomized study including 44 TBI patients reported that Hb separation between these two strategies was feasible and resulted in less RBCT in the restrictive group [18].

## Objectives

Our primary research question is to determine whether a “liberal” strategy of maintaining Hb concentrations at 9 g/dL or higher would result in a different neurological outcome when compared to a “restrictive” approach to RBC transfusion maintaining Hb concentrations at 7 g/dL or higher in critically ill patients with anemia (Hb ≤ 9 g/dL) and acute brain injury. We expect fewer complications in the restrictive group but an improvement in brain oxygenation in the liberal group, with a potential impact on neurological outcomes.

## Trial design

We will perform a prospective, multicenter phase 3, two-arm, randomized, investigator-initiated, superiority, pragmatic, and open-label study in anemic (i.e., Hb ≤ 9 g/dL) patients with an acute brain injury. Allocation is in a 1:1 ratio.

## Methods: participants, interventions, and outcomes

### Study setting

This study will be conducted in 72 intensive care units (ICUs) worldwide. The complete list of recruiting sites is shown in Supplemental Table 1. The selection of participating ICUs was initiated within the Neuro-Intensive Care (NIC) Section of the European Society of Intensive Care Medicine (ESICM), with the identification of national investigators who then selected potential academic and non-academic ICUs with neurosurgical facilities and an adequate number of patients (> 50) with acute brain injury admitted per year. The study was initially expected to last 4 years, assuming the inclusion of 75–95 patients/month in 50–70 different centers. The final duration of the study will be almost 7 years, because of the slow recruitment rate, which is in part due to the recent pandemic crisis.

**Table 1:** Inclusion and exclusion criteria of the TRAIN study

Inclusion criteria	Exclusion criteria
TBI, SAH, or ICH	Other neurological diseases, such as ischemic stroke or post-anoxic coma; status epilepticus without underlying brain injury; CNS infections (community-acquired; hospital-acquired; ventriculitis; post-operative)
Age ≥ 18 and ≤ 80 years	
GCS ≤ 13 on randomization	Known previous neurological disease, causing significant cognitive and/or motor handicap
Expected ICU stay > 72 h	ICH due to AVM or brain tumor
Hb ≤ 9 g/dL within 10 days from brain injury	Inability (religious reasons) or reduced ability (lack of compatible blood) to receive blood products
	Active and uncontrolled bleeding at the time of enrollment
	GCS of 3 with both pupils fixed and dilated; brain death or imminent death (within 24 h)
	Pregnancy
	Medical need to correct anemia (e.g., active coronary disease or severe cardiac disease) with target Hb levels > 9 g/dL
	DNE orders
	Previous allo-immunization due to transfusion, limiting RBC availability

*GCS*, Glasgow Coma Score; *ICU*, intensive care unit; *Hb*, hemoglobin; *DNE*, do-not-escalate (i.e., vasopressors, mechanical ventilation, renal replacement therapy); *CNS*, central nervous system; *ICH*, intracranial hemorrhage; *AVM*, arterio-venous malformation; *TBI*, traumatic brain injury; *SAH*, subarachnoid hemorrhage

### Eligibility criteria

All patients admitted to the ICU with TBI, SAH, or intracerebral hemorrhage (ICH, either primary or anticoagulant-associated) are screened for study eligibility within the first 10 days after the initial injury. Patients are eligible regardless of their need for surgical intervention or RBCT for acute bleeding. Anemia is defined as Hb ≤ 9 g/dL and should occur within this time period. If a patient is considered as not eligible, the reason is recorded as for eligible patients who decide not to participate. No other specific information is recorded for such patients. Inclusion and exclusion criteria are shown in Table 1 and are checked by the attending physician and confirmed by the local investigator.

### Who will take informed consent?

Research nurses or local investigators will introduce the study to the legal representatives (i.e., patient’s next of kin, legal guardian) explaining the main issues of the trial. All aspects of the trial will be discussed with the legal representatives and an informed discussion with be undertaken. Research nurses or local investigators will obtain written consent from legal representatives willing to let the patient participate to the trial. A copy of the informed consent will be given to the legal representative. The information and consent forms are part of a unique document. Small changes might happen to this procedure, according to legal requirements in different countries.

### Provisions for post-trial care

There is no anticipated harm and compensation for trial participation, since the transfusion thresholds that will be compared are already variably used in routine care.

### Study outcomes

The primary outcome measure is the proportion of patients with unfavorable neurological outcome at 180 days after randomization, assessed using the eGOS and dichotomized as “unfavorable” (eGOS 1–5) or “favorable” (eGOS 6–8). This scale has been largely used to assess neurological outcome in different interventional randomized trials dealing with acute brain-injured patients; mortality is part of the scale (eGOS 1).

Secondary outcome measures are (1) 28-day survival; (2) eGOS distribution between the two groups (i.e., ordinal outcome analysis); (3) ICU and hospital lengths of stay; (4) presence and severity of extra-cerebral organ dysfunction/failure, assessed using the daily Sequential Organ Failure Assessment (SOFA) score; (5) infection rate, except those involving only the central nervous system (CNS); (6) composite outcome (death and/or organ dysfunction/failure); (7) fluid balance; and (8) for centers monitoring brain tissue oxygenation (PbtO_2_), time spent with P_bt_O_2_ < 20 mmHg (brain hypoxic burden) will also be collected and analyzed, but reported into a separate secondary analysis.

### Participant timeline

**Fig. 1:**
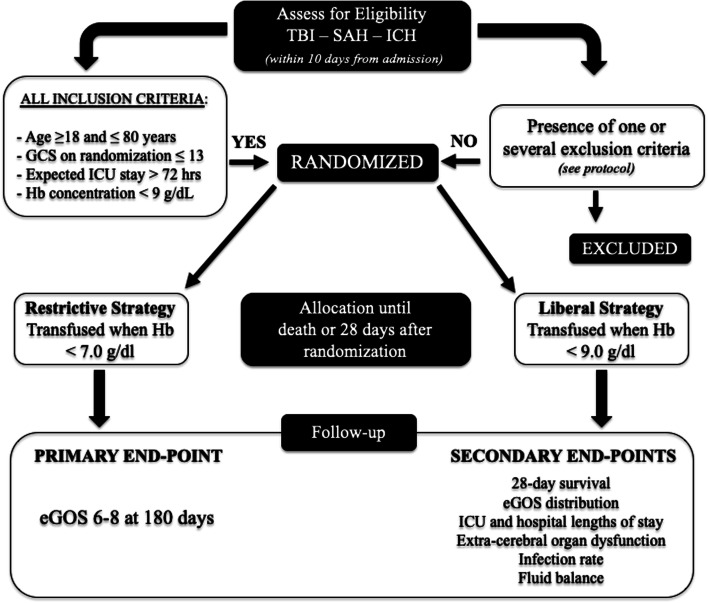
Study flow diagram. Hb, hemoglobin; TBI, traumatic brain injury; SAH, subarachnoid hemorrhage; ICH, intracerebral hemorrhage; GCS, Glasgow Coma Scale; ICU, intensive care unit; eGOS, extended Glasgow Outcome Scale

The participant timeline is reported in Fig. 1.

### Sample size

The primary outcome measure of this study is the occurrence of unfavorable neurological outcome at 180 days, evaluated by a eGOS of 1–5. To calculate the sample size, we estimated a mortality rate of 15% and a percentage of poor neurological outcome of 35% (i.e., eGOS 1–5 of 50%). There are no specific epidemiological data to support this assumption, as the population is mixed (i.e., TBI, SAH, and ICH) and the proportion of patients being eligible for the study remains unknown. Thus, the initial sample size calculation identified a total of 2095 patients needed to be recruited for each arm to achieve a power of 90% at a two-sided alpha level of 0.05 or less to detect a reduction of poor outcome rate at 180 days from 50 to 45% (absolute reduction of 5%, i.e., 10% relative reduction) in one of the two arms. Sample size calculation was adjusted twice. The first change (approved in August 2019) consisted in considering a reduction in unfavorable neurological outcome rate at 180 days from 50 to 40% (absolute reduction of 10%, i.e., 20% relative reduction, *n* = 1000) in one of the two arms, considering the relatively low number of patients being eligible for the study. The second change (approved in June 2022) consisted in considering a reduction in unfavorable neurological outcome rate at 180 days from 50 to 39% (absolute reduction of 11%, i.e., 22% relative reduction, *n* = 756–378 patients per group) in one of the two arms, with a power of 85% and at a two-sided alpha level of 0.05. Considering a potential 5% lost to follow-up, a total of 794 patients (397 per group) are needed to complete the study. This last modification was decided because of the coronavirus disease 2019 (COVID-19) pandemic, which significantly disrupted recruitment and blood availability and led to uncertainty over future recruitment.

### Recruitment

All adult ICU patients with a TBI, SAH, or ICH and having an Hb value of 9 g/dL or less within the first 10 days since admission can be considered for enrolment; they will be included and randomized by local investigators. The estimated recruitment is 0.1–0.2 patient per center per month, within an expected recruitment period of 6 years; however, this estimation is largely influenced by the COVID-19 pandemic, the active participation of each center, and the different timing of ethics approval in all countries.

## Methods: assignment of interventions

### Assignment of interventions: allocation

## Sequence generation

All eligible acute brain-injured patients during the study period in one of the participating centers can be considered for enrolment in the study. Randomization is performed using a computer-generated random sequence (variable blocks of 4, 6, and 8), stratified by center, by disease (TBI, SAH, or ICH), and by the Glasgow Coma Scale (GCS) at the moment of randomization (3–5; 6–9; 10–13).

## Concealment mechanism

Randomization will be performed using the online eCRF; if the subject meets all inclusion and exclusion criteria, the investigator will include some baseline characteristics and the randomization treatment arm will be provided by the web-based system (ClinFile, https://clinfile.com).

## Implementation

Investigators of all participating centers can sign into the web-based randomization system and randomize their patients. Patients will be automatically allocated to one of the treatment arms by the system.

## Assignment of interventions: who will be blinded?

Neither ICU nor hospital personnel is blinded to the treatment assignment, because patients are easily distinguishable by routine daily assessment of Hb concentrations. However, the final neurological evaluation of the patient is performed by assessors who are blinded to the group assignment.

### Procedure for unblinding if needed

As the study is open-label, no indications for breaking the randomization code are provided in the protocol. Randomization is communicated from the local principal investigator of each participating hospital to the study team. Based on the Hb daily levels achieved, concealed allocation can be controlled.

## Methods: data collection and management

### Plans for assessment and collection of outcomes

Data collection on admission will include the following: demographic characteristics, comorbidities, source of admission, primary and secondary admission diagnoses, Acute Physiologic Assessment and Chronic Health Evaluation (APACHE) II score (the worst values of the first 24 h), Sequential Organ Failure Assessment (SOFA) score on admission, GCS immediately after injury, GCS on hospital admission after initial resuscitation, initial Hb concentration, and sodium and glucose levels.

For patients with TBI, the following data are also collected: Marshall score on cerebral CT scan (the worst of the first 24 h), presence of traumatic SAH and/or epidural mass on CT scan, pupillary reactivity, mechanisms of injury, hypoxemia (defined as a SpO_2_ < 90% for more than 5 min with or without endotracheal intubation and under oxygen therapy) and/or hypotension (defined as a systolic blood pressure < 90 mmHg for more than 5 min despite fluid resuscitation) before or on hospital arrival, intracranial pressure (ICP) monitoring within the first 48 h, and previous therapy with antiplatelet drugs or anticoagulants.

For patients with SAH, the following data are also collected: World Federation of Neurological Society (WFNS) score; Fisher CT-scan scale; ICP monitoring within the first 48 h; pupillary reactivity on arrival; hydrocephalus; diagnosis of vasospasm (either using trans-cranial Doppler [TCD], contrast CT scan, and/or angiography); development of delayed neurological ischemic deficit (DNID); therapies for DNID; and occurrence of DCI. TCD-vasospasm is defined as a mean flow velocity in any vessel > 200 cm/s or > 120 cm/s and a Lindegaard ratio > 3 [22]. Angiographic or contrast CT-scan vasospasm is defined by a neuroradiologist as moderate-to-severe arterial narrowing (> 50%) on specific imaging not attributable to atherosclerosis, catheter-induced spasm, or vessel hypoplasia. Definition of DNID is based on the development of new focal neurological signs, deterioration in the level of consciousness, or both, when the cause is felt to be ischemia attributable to vasospasm after other possible causes of worsening (e.g., hydrocephalus, seizures, metabolic derangement, infection, or excessive sedation) have been excluded [23]; as such, DCI is defined as the appearance of DNID and/or a new infarction on cerebral CT scan or magnetic resonance imaging (MRI), when the cause was attributed to vasospasm [23].

For patients with ICH, the following data are collected: ICH volume > 30 mL on the initial CT scan (measured/estimated by local neuroradiologist); the presence of intra-ventricular hemorrhage; location (deep, cortical, infratentorial); ICP monitoring within the first 48 h; and pupillary reactivity on arrival.

Daily data collection (only during the ICU stay) includes daily GCS score; Hb concentration (minimum and maximum); sodium and glucose levels at 8 am (or the first value of the day); ICP levels at 8 am (or the first value of the day); maximum ICP levels during the day; SOFA score; the presence of infection (location, pathogen, treatment); the presence of sepsis; the occurrence of serious adverse events (*see paragraph on adverse events*); the need for second-tier therapies for increased ICP (hypothermia; barbiturates; decompressive craniectomy) or seizures (convulsive or non-convulsive); and daily fluid intake and urine output (to calculate daily fluid balance).

Data on each transfused RBC unit during the ICU stay and premature study termination (documenting the reason and time of termination) are recorded. In case of death, reasons for withdrawal of care are collected. ICU and hospital lengths of stay, the duration of mechanical ventilation over the first 28 days (either by endotracheal tube or tracheostomy), the need for tracheostomy on ICU discharge, and location of discharge after a hospital stay (home vs. rehabilitation vs. nursing home) are recorded. Follow-up at 180 days for primary outcome measure assessment is performed.

Data will be collected and stored as described at point 19, into the eCRF. After 6 months, patients who are still alive will be contacted by phone or by face-to-face meeting, according to local practice, for a structured interview to obtain the eGOS scale. The eGOS is widely used to assess neurological outcome in this setting [19].

### Plans to promote participant retention and complete follow-up

As crossover to another treatment arm than allocated is allowed, the crossover will be mentioned in the eCRF. In case of no informed consent, the patient will no longer be exposed to the allocated study intervention and the patient will be withdrawn from the study. As the eGOS assessment is recorded by a structured telephone or face-to-face interview, several attempts will be made to contact those patients not responding to the phone or not coming to scheduled visits; the general practitioner (GP) of the patient can also be contacted to facilitate outcome assessment.

### Plans for collection, laboratory evaluation, and storage of biological specimens for genetic or molecular analysis in this trial/future use

See also the “Additional consent provisions for collection and use of participant data and biological specimens” section; there will be no biological specimens collected.

### Data management.

For each randomized patient, an eCRF will be generated. Central data management will be performed using a dedicated website (https://train-esicm.clinfile.com) by the research team and data managers of the trial coordination center. Trial data will be entered in the patient’s eCRF by the local investigator or research nurse. The eCRF gives multiple tools and checkpoint to promote data quality (i.e., avoid errors in the biological variable report). The project leader will screen the website regularly for missing and incorrect data. When necessary, the project leader will contact the local investigator to adjust or complete the eCRF. The data will be kept for at least 10 years.

### Confidentiality

All randomized patients are identified by the center number followed by the patient number (i.e., 01 + 001 = 01/001). Trial personnel will not distribute patients’ names outside the local hospital. On screening forms, eCRF, and all other study documents, patients will never be identified by their names, but only by their randomization numbers. The subject identification code will be safeguarded by the local investigator.

### Statistical methods: outcomes

Analysis of data is based on intention-to-treat. Statistical analysis will be performed using the last version of SPSS for Windows (Chicago, USA). Continuous variables will be summarized using medians and quartiles and analyzed using a Wilcoxon rank sum test. Categorical variables will be analyzed using the Fisher exact test. The primary outcome comparisons will be analyzed using a chi-square analysis and reported as an absolute risk reduction of poor outcome and its corresponding 95% confidence interval. The primary outcome will also be adjusted for pre-specified covariates (stratification criteria) and presented for each category of brain injury (TBI, SAH, ICH).

All secondary outcomes will be analyzed using independent samples *t*-tests, chi-square test, as appropriate, without additional adjustment. For 28-day mortality, the Cox proportional hazard model will be used to determine time-to-event hazard ratios and 95% confidence intervals.

### Statistical methods: additional analyses

Subgroup analyses will be performed according to (a) underlying brain injury (TBI vs. SAH vs. ICH); (b) GCS at the moment of randomization (3–5; 6–8; 9–11); (c) presence of increased intracranial hypertension (defined as the need for specific therapies to reduce intracranial pressure [ICP]—if no ICP monitoring, then the patient is considered as not having intracranial hypertension); (d) age (< 45 years or ≥ 45 years); and (e) SOFA on randomization (< 8 and ≥ 8). Post hoc analyses will include (a) high- vs. middle- to low-income countries and (b) high-recruiting (i.e., > 25 patients) centers vs. others. All analyses will be processed by an independent statistician.

### Statistical methods: analysis to handle protocol non-adherence and any statistical methods to handle missing data

No specific analysis will be provided using the “per protocol” or “as treated” analysis set. Given our expectation that very few patients will crossover or be lost to follow-up, these analyses should agree very closely with the intention-to-treat analysis. No imputation will be performed for missing outcome data.

### Interim analysis

Initially, two interim analyses (i.e., after 200 and 700 patients) were scheduled; however, one interim analysis at 300 patients was therefore decided to assess for protocol compliance, main outcome, and SAEs, according to data collection completeness. An independent statistician, who has access to the whole database, reported these data to the DMSC by maintaining the blinding for the study groups (i.e., group A vs. group B). After the analysis of this report, the DMSC sent a report to continue the study recruitment without any safety issue.

### Statistical methods: plans to give access to the full protocol, participant-level data, and statistical code

No later than 3 years after the collection of the primary outcome of the last included patient, a completely deidentified dataset will be shared into an appropriate data archive for any scientific and reproducibility purposes.

## Methods: oversight and monitoring

### Composition of the coordinating center and trial steering committee

The principal investigator will have the responsibility for the study management. The study executive committee consists of the principal investigator, project coordinator, and research nurse, who are all affiliated to the Hôpital Erasme in Brussels (Belgium). The executive committee will be responsible for the daily running of the trial. The trial steering committee consists of the member of the Neuro-Intensive Care Section of ESICM, who meet twice a year (except during the pandemic) to have an update of the study.

### Composition of the data safety monitoring committee, its role, and reporting structure

The study is considered as a low-risk study; a Data Monitoring Safety Committee (DMC) has been created to perform the interim analyses for safety, futility, or efficacy so that the executive and steering committee can remain blinded for the study outcomes. All the DMSC members (Supplemental Table 2) have no conflict of interest with the sponsor of the study and are not involved in the study. DMSC membership is for the duration of the clinical trial. The DMSC has therefore monitored inclusion rate, losses to follow-up, or potential harms, making the recommendation that the trial continues to recruit participants or whether recruitment should be terminated earlier.

**Table 2:** Serious adverse event (SAE) of the TRAIN study

SAE	Definition
Severe hypertension	Mean arterial pressure (MAP) > 130 mmHg for more than 1 h and needing active therapy, in the absence of vasopressor agents and increased intracranial hypertension
Severe hypotension	MAP < 65 mmHg for at least > 1 h, not responding to fluid therapy and needing vasopressor therapy, in the absence of active bleeding
Venous thrombotic events	Deep vein thrombosis (formation of a blood clot within a deep vein in the legs or arms, which may be associated with non-specific signs, such as pain, swelling, redness, warmness, and engorged superficial veins—it can be diagnosed either by echography, venography, or CT imaging); pulmonary embolism (formation of a clot within the pulmonary arterial circulation, diagnosed by contrast pulmonary CT scan or echocardiography)
Acute myocardial ischemia	Acute myocardial infarction (ST-elevation and non-ST-elevation myocardial infarction) or unstable chest pain diagnosed during current hospital admission, according to specific criteria (i.e., elevated biomarkers of myocardial injury, ischemic signs on ECG, clinical suspicion) and the patient has received specific treatment (reperfusion strategies such as percutaneous coronary intervention [PCI] or thrombolysis) or initiation/increase of antithrombotic drug treatment during current ICU admission
Intestinal ischemia	Ischemic lesions confirmed by endoscopy and/or open surgery
Acute peripheral limb ischemia	Clinical signs and the need for open or percutaneous vascular intervention, amputation, or initiation/increased antithrombotic treatment
Anaphylactic reaction to RBC transfusion	Muco-cutaneous signs (i.e., urticaria, pruritus) and/or hemolytic anemia within 24 h after transfusion
ARDS	Acute hypoxemia with bilateral infiltrates, according to recent definitions
TRALI	ARDS occurring within 6 h after RBC transfusion
TACO	Acute hypoxemia (PaO_2_/FiO_2_ < 300 regardless of positive end-expiratory pressure [PEEP]) with bilateral lung infiltrates and occurrence within 6 h after RBC transfusion and increased blood pressure and positive fluid balance
Sepsis	Presence of an infection and organ failure attributed to it. Septic shock = arterial hypotension (MAP < 65 mmHg) despite adequate fluid resuscitation and necessitating vasopressor therapy
Infections	According to CDC definitions
Brain tissue hypoxia	For those patients with P_bt_O_2_ monitoring, values of < 20 mmHg for at least 1 h over the last 24 h

### *Adverse event* reporting and harms

A complete list of serious adverse events (SAEs) is presented in Table 2. As expected, mortality is relatively high in this patient population; therefore, death will not be reported as SAE. SAEs are chosen based on possible relation with blood transfusions.

Each type of SAE and the total number of SAE is reported in the eCRF and do not have to be reported separately. The annual report of SAEs is communicated to the Ethics Committee of the trial coordinator center, as a responsibility of the study coordinator and the primary investigator. As the interim analysis did not report potential harm differences between groups, the SAE report to the Data Safety Monitoring Committee (DSMC) was decided only on the basis of the annual evaluation of the Ethics Committee.

### Frequency and plans for auditing trial conduct

Because of the number of recruiting centers worldwide and the lack of funding, monitoring has been delegated to the national investigator, according to local policies. Monitoring will be performed by an independent and qualified monitor; visits to each center will be organized according to national requirement, to ensure patients’ rights and safety, as well as compliance to the protocol, adequate data collection, and outcome reporting. Data monitoring will consider the inclusion rate, informed consent procedures, trial and investigators’ main documents and files, endpoints and SAE report, and source data (i.e., hospital medical records, medical notes, laboratory findings, electronic data).

verification. Findings from the monitoring visits will be reported by the monitor to the national investigator through a specific report. Considering the variability in resources and monitoring availability, it has been estimated to have a complete monitoring on at least 40% of recruited patients.

### Plans for communicating important protocol amendments to relevant parties

Relevant protocol modifications (i.e., protocol amendments) will be communicated to relevant parties (i.e., national investigators, ethics committees, study insurance, trial registry, ClinicalTrials.org) by the principal investigator via emails. A list of all participating investigators is also available on the study website to send regular communications.

## Ethics and dissemination

### Research ethics approval

The “Comite d'Ethique Erasme-ULB” approved this multicentric study on the 14th of March 2016 (P2015/327) and ensured that written informed consent to participate will be obtained from all participants. The first approval included also all participating centers in Belgium. All documents related to the study and the approval from the “Comite d'Ethique Erasme-ULB” was therefore sent to all national investigators to ensure ethics approval according to National Laws in the countries of the trial sites. No deviation from the protocol has been implemented without the prior review and approval of the ECs.

### Informed consent

Written informed consent must be obtained prior to the randomization to the study. Because of the difficulties predicting the exact moment at which anemia may occur during the ICU stay, it is recommended that written informed consent be obtained as soon as possible after ICU admission so that randomization can be performed rapidly when the inclusion criteria are met. For patients who are unable to consent, their legal representatives are informed of the study as soon as possible and must sign for participation in the trial before randomization. Subjects with sufficient neurological recovery are informed of their study participation and be asked to provide their consent for the use of their data. Patients or next of kin can withdraw consent at any time during the study without giving a specific reason. Withdrawal should not influence the standard of care, with an Hb threshold for RBC transfusion that will then be decided by the attending physician. Patients withdrawing from the study are asked to consent for the inclusion of data collected before withdrawal. The site investigator may also withdraw a subject from the study for safety reasons (e.g., acute myocardial infarction needing higher Hb levels).

### Additional consent provisions for the collection and use of participant data and biological specimens

No biological specimen will be collected*.*

### Access to data

The protocol of the study is available under request at the Ethics Committee of the Hôpital Erasme, Brussels, Belgium. The dataset generated during this study will be available from the corresponding author upon reasonable request.

### Dissemination policy: trial results

At the end of the outcome assessment collection, data will be analyzed within 2 months and presented to all investigators before publication. Important steps will be to disseminate the obtained results among health care professionals, patients, and policymakers in order to help in improving clinical practice in this setting. The project group members have a broad network in Europe and are well-positioned to guarantee dissemination of the study results obtained among their colleagues, by presenting the results at national and international podia and by writing reports and papers. Upon trial completion, the main manuscript will be submitted to one of the major clinical journals and the results will also be available after acceptance and publication at the TRAIN trial homepage.

### Dissemination policy: authors’ contribution

All centers that have at least 30 patients recruited will earn an authorship in the “authors’ list”; a second author will be allowed for each 30 additional patients recruited. The national investigator will also be part of the authors’ list if the total number of patients randomized in their country exceeds 30. The author list will take into account the number of enrolled patients; FS Taccone and JL Vincent will be the first and last authors. For other co-authors, in case of a similar number of recruited patients, the participation to data analysis and contribution to the manuscript will be considered for the order authorship.

## Discussion

This trial will assess the impact of two strategies of determining when to administer a blood transfusion in a large cohort of critically ill patients with a primary acute brain injury. The results of this trial will help to improve blood product and transfusion use in this specific patient population and will provide additional data in some subgroups of patients at high risk of secondary brain injury, such as those with intracranial hypertension or cerebral vasospasm.

Isovolemic anemia (i.e., Hb of 5 g/dL) induced in healthy volunteers resulted in some alterations in memory and motor skills [20]; however, these Hb levels are not currently recommended in critically ill patients. Interestingly, in acute brain injury, the Hb threshold associated with potential cerebral hypoxia may be higher than in the healthy brain, because of the exhausted cerebrovascular reserve, i.e., cerebral vasodilation that adapts cerebral blood flow to tissue oxygen demand [21]. As such, our primary research question will focus on the comparison between a restrictive, which is actually used in most critically ill patients, and a liberal transfusion policy in a patients’ population where no solid evidence is available. Two other ongoing studies (NCT03260478 and NCT03309579), conducted in TBI and SAH patients, respectively, will be also completed shortly and potentially provide additional findings on this topic. Also, future individual patient meta-analyses including all randomized subjects in these studies could also help to identify the subgroup of patients who might benefit the most from one or another transfusion strategy.

We have selected Hb values to trigger RBCT, as this is commonly used in clinical practice. However, an absolute Hb level would not provide relevant information on the patient’s tolerance to anemia, which is highly dependent on the volume status of the patient, physiological reserve, and the dynamics of the anemia (i.e., acute vs. chronic) [22]. Moreover, Hb values would not consider other phenomena (i.e., blood flow redistribution to the heart and the brain, altered microcirculation, baseline tissue metabolism) that are all important for the tolerance of severe degrees of normovolemic anemia from healthy and injured subjects [23, 24]. Despite all these limitations, the findings from this study will have a direct impact on transfusion management, which is largely based on Hb measurements.

The strengths of this study are the multicenter design of the study, the easy-to-implement protocol, the lack of additional therapeutic interventions besides standard care which will improve protocol adherence, and the inclusion of several acutely brain-injured patients (i.e., TBI, SAH, and ICH). Some limitations need also to be acknowledged. First, brain injury could occur also at higher Hb values; however, this will not be assessed in this study. Second, separation of Hb values between groups over time will not be very large [22] and one may argue that this would not be sufficient to show significant differences in outcome between the two transfusion strategies. Third, the availability of blood as well as blood storage time and quality will be dependent from local organization and participating centers.

Whether this could potentially influence the outcome of our analysis remains an unmeasured confounder, even if the outcome assessment will be adjusted to the recruiter center. Finally, the revised sample size may increase the risk to overlook a potentially clinically relevant difference in the primary outcome.

### Trial status

The TRAIN study is currently recruiting in 72 hospitals worldwide; recruitment began in September 2016 and will be completed on the 31th of December 2022. To date, 791 participants have been recruited. During the COVID-19 pandemic, fewer participants than expected have been recruited, which has brought to a recalculation of the sample size. The planned end of data collection is June 2023 and data presentation is expected in late 2023.

## Supplementary Information

**Additional file 1: Supplemental Table 1.** List of participating centers.

**Additional file 2.** SPIRIT Checklist for Trials.

## Data Availability

Any data required to support the protocol can be supplied on request.

## Abbreviations

APACHE II: Acute Physiology and Chronic Health Evaluation
ARDS: Acute respiratory distress syndrome
AVM: Arterio-venous malformation
CNS: Central nervous system
CRF: Case report form
DSMC: Data and Safety Monitoring Committee
EC: Ethics Committee
ESICM: European Society of Intensive Care Medicine
GCS: Glasgow Coma Scale
eGOS: Extended Glasgow Outcome Scale
Hb: Hemoglobin
ICH: Intracranial hemorrhage
ICP: Intracranial pressure
ICU: Intensive care unit
mRS: Modified Rankin scale
NCS: Neurocritical Care Society
P_bt_O_2_: Brain tissue partial pressure of oxygen
PI: Principal investigator
RBC: Red blood cells
SAE: Serious adverse event
SAH: Subarachnoid hemorrhage
SIZ: Belgian Society of Intensive Care
SOAP: Sepsis Occurrence in Acutely Ill Patients
SOFA: Sequential Organ Failure Assessment
TACO: Transfusion-associated circulatory overload
TBI: Traumatic brain injury
TRALI: Transfusion-related acute lung injury
