# Correction: TRansfusion strategies in Acute brain INjured patients (TRAIN): a prospective multicenter randomized interventional trial protocol

**DOI:** 10.1186/s13063-024-08282-8

**Published:** 2024-06-23

**Authors:** Fabio Silvio Taccone, Rafael Badenes, Carla Bittencourt Rynkowski, Pierre Bouzat, Anselmo Caricato, Pedro Kurtz, Kirsten Moller, Manuel Quintana Diaz, Mathieu Van Der Jagt, Walter Videtta, Jean-Louis Vincent

**Affiliations:** 1 Department of Intensive Care, Route de Lennik, Erasme Hospital, Université Libre de Bruxelles, 808, 1070 Brussels, Belgium; 2 Hospital Nacional Professor Alejandro Posadas, Buenos Aires, Argentina; 3 Department of Anesthesiology and Surgical-Trauma ICU, Hospital Clínic Universitari de Valencia, University of Valencia, Valencia, Spain; 4 Intensive Care Unit of Cristo Redentor Hospital, Porto Alegre, Brazil; 5 Intensive Care Unit, Hospital Ernesto Dornelles, Porto Alegre, Brazil; 6 Université Grenoble AlpesInserm, U1216, CHU Grenoble Alpes, Grenoble Institut Neurosciences, Grenoble, France; 7 Institute of Anesthesiol- Ogy and Intensive Care, Catholic University School of Medicine, Rome, Italy; 8 Department of Intensive Care Medicine, DOr Institute of Research and Edu- Cation, Rio de Janeiro, Brazil; 9 Department of Neurointensive Care, Instituto Estadual Do Cerebro Paulo Niemeyer, Rio de Janeiro, Brazil; 10 Department of Neuroanaesthesiology, Neuroscience Centre, Copenhagen University, Hospital Rigshospitalet, Copenhagen, Denmark; 11 Department of Clinical Medicine, Faculty of Health and Medical Sciences, University of Copenhagen, Copenhagen, Denmark; 12 Department of Intensive Care Medicine, Hospi- Tal Universitario de La Paz, Madrid, Spain; 13 Department of Intensive Care Adults, Erasmus MC – University Medical Center Rotterdam, Rotterdam, The Netherlands

**Correction** **:** **Trials 24, 20 (2023)**

**https://doi.org/10.1186/s13063-022-07061-7**

Following the publication of the original article [1], the authors noticed two errors in the Methods section, which became relevant during data analyses. In the sub-section “Statistical Analysis”, it was stated that subgroup analyses will be performed according to Glasgow Coma Scale (GCS) at the moment of randomization (3-5; 6-8; 9-11). However, this is in contrast with the “Sequence generation” sub-section, in which all eligible acute brain injured patients underwent randomization which was also stratified by the GCS at the moment of randomization (3-5; 6-9; 10-13). Moreover, the initial proposal would exclude some patients (e.g. GCS 12 or 13) from the subgroup analysis. This should read: “Subgroup analyses will be performed according to … b) GCS at the moment of randomization (3-5; 6-9; 10-13); …”. The second error is the lack of cerebral ischemia among serious adverse events, which was present in the protocol submitted for Ethical Approval. The definition of cerebral ischemia was “New (i.e. after randomization) ischemic lesion visible on brain imaging (i.e. either CT-scan or MRI).”
