# Supplementary material for: TRansfusion strategies in Acute brain INjured patients (TRAIN): a prospective multicenter randomized interventional trial protocol
Source: Trials. 2023 Jan 7;24:20. doi: 10.1186/s13063-022-07061-7 (PMC9825124; doi:10.1186/s13063-022-07061-7)
Supplement: Supplementary file 1 — Additional file 1: Supplemental Table 1. List of participating centers. [file 13063_2022_7061_MOESM1_ESM.docx]

**Supplemental Table 1.** List of participating centers.

| **Site** | **Institution** | **City, Country** |
| --- | --- | --- |
| [**01/03**](https://train-esicm.clinfile.com/Modules/crf/liste_patients.php?tri_liste=patient&ec_id=75) | HZGA Balestrini | Buenos Aires, Argentina |
| [**01/02**](https://train-esicm.clinfile.com/Modules/crf/liste_patients.php?tri_liste=patient&ec_id=74) | HIGA Diego Paroissien | Buenos Aires, Argentina |
| [**01/07**](https://train-esicm.clinfile.com/Modules/crf/liste_patients.php?tri_liste=patient&ec_id=121) | Terapia intensive Hopital El Cruce | Buenos Aires, Argentina |
| [**01/01**](https://train-esicm.clinfile.com/Modules/crf/liste_patients.php?tri_liste=patient&ec_id=73) | HZGA Simplemente Evita | Buenos Aires, Argentina |
| [**01/05**](https://train-esicm.clinfile.com/Modules/crf/liste_patients.php?tri_liste=patient&ec_id=77) | Hospital Nacional Profesor Dr. Alejandro Posadas | Buenos Aires, Argentina |
| [**01/08**](https://train-esicm.clinfile.com/Modules/crf/liste_patients.php?tri_liste=patient&ec_id=123) | HCAS Cuenca Alta | Buenos Aires, Argentina |
| [**01/06**](https://train-esicm.clinfile.com/Modules/crf/liste_patients.php?tri_liste=patient&ec_id=78) | Hospital Artemides Zatti | Viedma, Argentina |
| [**01/04**](https://train-esicm.clinfile.com/Modules/crf/liste_patients.php?tri_liste=patient&ec_id=76) | HCM Juan Sanguinetti | Buenos Aires, Argentina |
| [**02/07**](https://train-esicm.clinfile.com/Modules/crf/liste_patients.php?tri_liste=patient&ec_id=63) | Virga Jesse Ziekenhuis | Hasselt, Belgium |
| [**02/02**](https://train-esicm.clinfile.com/Modules/crf/liste_patients.php?tri_liste=patient&ec_id=58) | Cliniques Universitaires St-Luc UCL | Brussels, Belgium |
| [**02/06**](https://train-esicm.clinfile.com/Modules/crf/liste_patients.php?tri_liste=patient&ec_id=62) | AZ Sint Jan | Kortrijk, Belgium |
| [**02/03**](https://train-esicm.clinfile.com/Modules/crf/liste_patients.php?tri_liste=patient&ec_id=59) | UZ Leuven - Campus Gasthuisberg | Leuven, Belgium |
| [**02/11**](https://train-esicm.clinfile.com/Modules/crf/liste_patients.php?tri_liste=patient&ec_id=68) | AZ Delta | Roeselare, Belgium |
| [**02/12**](https://train-esicm.clinfile.com/Modules/crf/liste_patients.php?tri_liste=patient&ec_id=70) | CHU Ambroise Paré | Mons, Belgium |
| [**02/05**](https://train-esicm.clinfile.com/Modules/crf/liste_patients.php?tri_liste=patient&ec_id=61) | AZ Groeninge | Kortrijk, Belgium |
| [**02/01**](https://train-esicm.clinfile.com/Modules/crf/liste_patients.php?tri_liste=patient&ec_id=5) | Erasme University Hospital (ULB) | Brussels, Belgium |
| [**02/13**](https://train-esicm.clinfile.com/Modules/crf/liste_patients.php?tri_liste=patient&ec_id=125) | CHwapi | Tournai, Belgium |
| [**02/09**](https://train-esicm.clinfile.com/Modules/crf/liste_patients.php?tri_liste=patient&ec_id=65) | AZ Sint Dimpna | Geel, Belgium |
| [**02/04**](https://train-esicm.clinfile.com/Modules/crf/liste_patients.php?tri_liste=patient&ec_id=60) | UZ Gent-Ghent University Hospital | Gent, Belgium |
| [**02/08**](https://train-esicm.clinfile.com/Modules/crf/liste_patients.php?tri_liste=patient&ec_id=64) | AZ Turnhout | Turnhout, Belgium |
| [**06/04**](https://train-esicm.clinfile.com/Modules/crf/liste_patients.php?tri_liste=patient&ec_id=124) | Fundación Valle del Lili, University Hospital | Cali, Colombia |
| [**06/01**](https://train-esicm.clinfile.com/Modules/crf/liste_patients.php?tri_liste=patient&ec_id=14) | Clinica Santagracia | Popayan, Colombia |
| [**06/03**](https://train-esicm.clinfile.com/Modules/crf/liste_patients.php?tri_liste=patient&ec_id=72) | Clinica Los Rosales | Pereira, Colombia |
| [**06/02**](https://train-esicm.clinfile.com/Modules/crf/liste_patients.php?tri_liste=patient&ec_id=71) | Clinica El Bosque | Bogota, Colombia |
| [**14/04**](https://train-esicm.clinfile.com/Modules/crf/liste_patients.php?tri_liste=patient&ec_id=67) | Policlinico Agostino Gemelli | Rome, Italy |
| [**23/01**](https://train-esicm.clinfile.com/Modules/crf/liste_patients.php?tri_liste=patient&ec_id=80) | Shohada Tabriz University of Medical Sciences | Tabriz, Iran |
| [**23/02**](https://train-esicm.clinfile.com/Modules/crf/liste_patients.php?tri_liste=patient&ec_id=81) | Sina Hospital | Tehran, Iran |
| [**24/01**](https://train-esicm.clinfile.com/Modules/crf/liste_patients.php?tri_liste=patient&ec_id=82) | Instituto Estadual do Cérebro Paulo Niemeyer | Rio de Janeiro, Brasil |
| [**25/01**](https://train-esicm.clinfile.com/Modules/crf/liste_patients.php?tri_liste=patient&ec_id=83) | Hospital Clinic Universitari de Valencia | Valencia, Spain |
| [**26/01**](https://train-esicm.clinfile.com/Modules/crf/liste_patients.php?tri_liste=patient&ec_id=84) | Hospital de Especialidades Eugenio Espejo | Quito, Ecuador |
| [**27/01**](https://train-esicm.clinfile.com/Modules/crf/liste_patients.php?tri_liste=patient&ec_id=85) | Conplejo Hospitalario de Leon | Leon, Spain |
| [**28/01**](https://train-esicm.clinfile.com/Modules/crf/liste_patients.php?tri_liste=patient&ec_id=86) | CHU Grenoble-Alpes | Grenoble, France |
| [**29/01**](https://train-esicm.clinfile.com/Modules/crf/liste_patients.php?tri_liste=patient&ec_id=87) | CHU Bicêtre | Paris, France |
| [**30/01**](https://train-esicm.clinfile.com/Modules/crf/liste_patients.php?tri_liste=patient&ec_id=88) | Hôpital Saint-Roch- CHU de Nice | Nice, France |
| [**31/01**](https://train-esicm.clinfile.com/Modules/crf/liste_patients.php?tri_liste=patient&ec_id=89) | CHU Nantes-Hôtel Dieu | Nantes, France |
| [**31/02**](https://train-esicm.clinfile.com/Modules/crf/liste_patients.php?tri_liste=patient&ec_id=117) | CHU Nantes-Hôpital Nord Laennec | Nantes, France |
| [**32/01**](https://train-esicm.clinfile.com/Modules/crf/liste_patients.php?tri_liste=patient&ec_id=90) | Hôpital Gabriel Montpied, CHU Clermont Ferrand | Clermont Ferrand, France |
| [**33/01**](https://train-esicm.clinfile.com/Modules/crf/liste_patients.php?tri_liste=patient&ec_id=91) | Hospital Universitario La Paz | Madrid, Spain |
| [**34/01**](https://train-esicm.clinfile.com/Modules/crf/liste_patients.php?tri_liste=patient&ec_id=66) | Hospital Ramón y Cajal | Madrid, Spain |
| [**35/01**](https://train-esicm.clinfile.com/Modules/crf/liste_patients.php?tri_liste=patient&ec_id=92) | Burnazian State Research Medical Center, Federal Medical | Moscow, Russia |
| [**36/01**](https://train-esicm.clinfile.com/Modules/crf/liste_patients.php?tri_liste=patient&ec_id=93) | Hospital San Bartolo | Quito, Ecuador |
| [**37/01**](https://train-esicm.clinfile.com/Modules/crf/liste_patients.php?tri_liste=patient&ec_id=94) | Centro Hospitalar Sao Joao | Porto, Portugal |
| [**38/01**](https://train-esicm.clinfile.com/Modules/crf/liste_patients.php?tri_liste=patient&ec_id=96) | CH Annecy Genevois | Annecy, France |
| [**39/01**](https://train-esicm.clinfile.com/Modules/crf/liste_patients.php?tri_liste=patient&ec_id=95) | Complejo Asistencial Universitario de Salamanca | Salamanca, Spain |
| [**40/01**](https://train-esicm.clinfile.com/Modules/crf/liste_patients.php?tri_liste=patient&ec_id=97) | Hospital Universitario y Politécnico de La Fe | Valencia, Spain |
| [**41/01**](https://train-esicm.clinfile.com/Modules/crf/liste_patients.php?tri_liste=patient&ec_id=98) | Hospital Universitario Puerta de Hierro | Majadahonda, Spain |
| [**42/01**](https://train-esicm.clinfile.com/Modules/crf/liste_patients.php?tri_liste=patient&ec_id=99) | Hospital Germans Trias i Pujol | Barcelona, Spain |
| [**43/01**](https://train-esicm.clinfile.com/Modules/crf/liste_patients.php?tri_liste=patient&ec_id=100) | Clínica la Merced | Baranquilla, Colombia |
| [**44/01**](https://train-esicm.clinfile.com/Modules/crf/liste_patients.php?tri_liste=patient&ec_id=101) | Hospital Cristo Redentor | Porto Alegre, Brasil |
| [**45/01**](https://train-esicm.clinfile.com/Modules/crf/liste_patients.php?tri_liste=patient&ec_id=102) | Hospital Moinhos de Vento | Porto Alegre, Brasil |
| [**46/01**](https://train-esicm.clinfile.com/Modules/crf/liste_patients.php?tri_liste=patient&ec_id=103) | Mechnikov Dnipropetrovsk Regional Clinical Hospital | Dnipro, Ukraine |
| [**47/01**](https://train-esicm.clinfile.com/Modules/crf/liste_patients.php?tri_liste=patient&ec_id=104) | Hôpital Militaire de Tunis | Tunis, Tunisia |
| [**48/01**](https://train-esicm.clinfile.com/Modules/crf/liste_patients.php?tri_liste=patient&ec_id=105) | Moscow Regional Clinical and Research Institute | Moscow, Russia |
| [**49/01**](https://train-esicm.clinfile.com/Modules/crf/liste_patients.php?tri_liste=patient&ec_id=106) | Tampere University Hospital | Tampere, Finland |
| [**50/01**](https://train-esicm.clinfile.com/Modules/crf/liste_patients.php?tri_liste=patient&ec_id=107) | Kuopio University Hospital | Kuopio, Finland |
| [**51/01**](https://train-esicm.clinfile.com/Modules/crf/liste_patients.php?tri_liste=patient&ec_id=108) | University Medical Centre Ljubljana | Ljubljana, Slovenia |
| [**52/01**](https://train-esicm.clinfile.com/Modules/crf/liste_patients.php?tri_liste=patient&ec_id=109) | Erasmus MC Universitair Medisch Centrum | Rotterdam, The Netherlands |
| [**53/01**](https://train-esicm.clinfile.com/Modules/crf/liste_patients.php?tri_liste=patient&ec_id=110) | Medical University Hospital of Lublin | Lublin, Poland |
| [**54/01**](https://train-esicm.clinfile.com/Modules/crf/liste_patients.php?tri_liste=patient&ec_id=111) | Thriasion General Hospital of Eleusis | Eleusina, Greece |
| [**55/01**](https://train-esicm.clinfile.com/Modules/crf/liste_patients.php?tri_liste=patient&ec_id=112) | Hamad General Hospital | Doha, Qatar |
| [**56/01**](https://train-esicm.clinfile.com/Modules/crf/liste_patients.php?tri_liste=patient&ec_id=113) | Cleveland Clinic Abu Dhabi | Abu Dhabi, EAU |
| [**57/01**](https://train-esicm.clinfile.com/Modules/crf/liste_patients.php?tri_liste=patient&ec_id=114) | Azienda Ospedaliera di Perugia | Perugia, Italy |
| [**58/01**](https://train-esicm.clinfile.com/Modules/crf/liste_patients.php?tri_liste=patient&ec_id=115) | Azienda Ospedaliera Santa Maria di Terni | Terni, Italy |
| [**59/01**](https://train-esicm.clinfile.com/Modules/crf/liste_patients.php?tri_liste=patient&ec_id=116) | Azienda Sanitaria Universitaria Integrata di Trieste, Hospital of Trieste | Trieste, Italy |
| [**60/01**](https://train-esicm.clinfile.com/Modules/crf/liste_patients.php?tri_liste=patient&ec_id=118) | Sanatorio Trinidad San IsidroSana | Martinez, Argentina |
| [**61/01**](https://train-esicm.clinfile.com/Modules/crf/liste_patients.php?tri_liste=patient&ec_id=119) | Sklifosovsky N.V. Research Institute of Emergency Medicine | Moscow, Russia |
| [**62/01**](https://train-esicm.clinfile.com/Modules/crf/liste_patients.php?tri_liste=patient&ec_id=120) | Dept. of Neuroanaesthesiology | Copenhagen, Denmark |
| [**63/01**](https://train-esicm.clinfile.com/Modules/crf/liste_patients.php?tri_liste=patient&ec_id=122) | HOME Hospital | Sao Paolo, Brasil |
| [**69/01**](https://train-esicm.clinfile.com/Modules/crf/liste_patients.php?tri_liste=patient&ec_id=126) | AMC | Amesterdam, The Netherlands |
| [**70/01**](https://train-esicm.clinfile.com/Modules/crf/liste_patients.php?tri_liste=patient&ec_id=127) | Tanta University Hospital | Tanta, Egypt |
| [**71/01**](https://train-esicm.clinfile.com/Modules/crf/liste_patients.php?tri_liste=patient&ec_id=128) | CHU Brest - La Cavale Blanche | Brest, France |
| [**72/01**](https://train-esicm.clinfile.com/Modules/crf/liste_patients.php?tri_liste=patient&ec_id=129) | Hospital del Mar, Universitat Pompeu Fabra | Barcelona, Spain |

**Supplemental Table 2.** Data Safety Monitoring Committee (DSMC)

Prof. Alain CARIOU

Department of Intensive Care

Hopital Cochin

Paris Descartes University

27, rue du Faubourg-Saint-Jacques

75014 Paris, France

email: alain.cariou@cch.ap-hop-paris.fr

Dr Christophe LELUBRE

Department of Internal Medicine

CHU Charleroi

Boulevard Zoé Drion, 1

6000 Charleroi, Belgium

email: christophe.lelubre@ulb.ac.be

Dr. Giuseppe RISTAGNO

Istituto di Ricerche Farmacologiche "Mario Negri" Milano

Via Giuseppe La Masa 19

20156 Milan, Italy

email: giuseppe.ristagno@marionegri.it
